# Microhabitat Selection for Overwintering: Overwintering Conditions of Three Jumping Spiders (*Pellenes tripunctatus, P. nigrociliatus*, and *Attulus penicillatus*) Living in Terrestrial Shells in the Czech Republic

**DOI:** 10.3390/insects13100950

**Published:** 2022-10-18

**Authors:** Kristína Dziváková, Zdeněk Faltýnek Fric, Vladimír Hula

**Affiliations:** 1 Department of Forest Ecology, Faculty of Forestry and Wood Technology, Mendel University in Brno, Zemědělská 3, 613 00 Brno, Czech Republic; 2 Department of Zoology, Fisheries, Hydrobiology and Apiculture, Mendel University in Brno, Zemědělská 1, 613 00 Brno, Czech Republic; 3 Biology Centre of the Czech Academy of Sciences, Institute of Entomology, Branišovská 31, 370 05 České Budějovice, Czech Republic

**Keywords:** steppe, Salticidae, habitat condition, spider behavior

## Abstract

**Simple Summary:**

We studied the overwintering ecologies of three endangered jumping spiders (*Pellenes tripunctatus*, *Pellenes nigrociliatus*, and *Attulus penicillatus*) in empty land-snail shells, specifically the microenvironmental factors determining their overwintering conditions, the presence of other species, and the presence of more than one specimen within a shell. We observed that the surrounding microhabitat was important for the occupancy of a shell during winter by a particular species. The females of *P. tripunctatus* and *P. nigrociliatus* preferred barren soil with the presence of pieces of herbs or grasses where they could hang the shell for egg laying, and as a retreat for the next generation. The interesting co-existence of several individuals of a single species was discussed.

**Abstract:**

Taking the regular overwintering of spider species in land snail shells as a model, we studied environmental conditions affecting the choice of overwintering sites in three jumping spider species: *Pellenes tripunctatus*, *Pellenes nigrociliatus*, and *Attulus penicillatus*. The research was conducted at 11 steppe localities on calcareous bedrock with abundant empty shells (mainly *Caucasotachea vindobonensis* and *Xerolenta obvia*). We documented 889 shells and collected 186 of them, of which 113 were inhabited by 146 spider individuals (13 species). Our three focal species made up 81.5% of these. We found different environmental preferences between the sexes in *P. tripunctatus* and *P. nigrociliatus*. These females preferred shells with more vegetation nearby. In the case of *P. tripunctatus*, these were shells with a higher proportion of herbs, whereas *P. nigrociliatus* selected for a higher proportion of moss. In the immediate vicinity of the shells, environmental conditions did not differ significantly. We found insufficient *A. penicillatus* to determine any preferences. We also recorded six overwintering *P. tripunctatus* individuals in a single shell (in environmental conditions preferred by females), consisting of five females and one male, which indicated an unusual social behavior for these spider species.

## 1. Introduction

Overwintering strategies are important for survival in temperate regions, and differences in shelter selection can be observed among sympatric or syntopic species. Shelter selection may vary within a single species of spider, such as between the sexes [1,2,3]. Very few studies, however, have dealt with environmental determinates of overwintering conditions.

A significant environmental preference for a specific microhabitat has, for example, been described in the jumping spiders *Psecas chapoda* and *P. viridipurpureus*, which show a strong association with plants in the families Bromeliaceae and Agavaceae that typically have rosette-shaped leaves. Interestingly, distribution models show differences between spiderlings and older individuals. Females with egg sacs occur closer to the central layer of the rosette, enabling spiderlings to reach shelter more quickly. Males usually occur on the top of the plants [4,5,6]. The jumping spider *Phidippus johnsoni* chooses relatively large stones or pieces of wood, wherein the animals create tubular nests at higher densities, especially during molting and oviposition. Males occur more frequently outside the nest, and their nests are not so dense [7]. Moreover, various nest associates have been found close to or directly inside spider nests [8]. Differences in the inhabitation of the host plant are visible in the species *Eris militaris*, which inhabits the upper and larger parts of the plant, while the smaller species *Synageles canadensis* inhabits the lower and smaller parts. Possible settlement by spiders is also determined by the health condition of the plant, and whether or not it is inhabited by other insects or spiders [9]. The jumping spider *Paraphidippus aurantius* uses the base of the needle clusters in *Pinus ponderosa* to build its nest. In addition, a certain fidelity or affinity to the nest has been found. Even after a nest is destroyed, the spider will return to the same place and repair the nest [10]. This behavior was also found in the previously mentioned *Phidippus johnsoni*. Females were observed in their nests for several months and oviposition was performed at the same nest repeatedly [7]. In the case of *Phidippus clarus*, even when that fidelity to the nest varied between months, females always oriented themselves according to a certain object that was near the nest [11]. In the case of *Gladicosa pulchra*, significant differences have been found in the inhabitancy of trees, and the males always inhabited the trees earlier than the females [12]. In the species *Frontinellina frutetorum* and *Neriene radiata*, height differences were found in the distribution of spiders on an inhabited conifer, as *F. frutetorum* always built its web in higher parts of the conifer [13]. Syntopic species *Geolycosa xera archboldi* and *Geolycosa hubbelli* also preferred differences in microhabitat conditions of vegetation [14]. The same was true for the syntopic species *Pardosa lugubris* and *Pardosa alacris* regarding the preference for density of the forest cover [15]. Depending upon where species live, they naturally choose the best and most advantageous hiding places that the habitats offer them. 

One of the most fascinating overwintering places for spiders consists of empty snail shells. Empty shells serve as important shelters for various species of invertebrates. Often, these are rare, endangered species, or species about which we have very little information [16,17]. Shells offer an advantageous microclimate that ensures both survival during hot summers (protection against drying) and protection from various predators. However, they are primarily inhabited when the air temperature begins to decrease. In general, spiders constitute the most common group of invertebrates using empty shells for hibernation. The most abundant inhabitants are spiders of the family Salticidae [16,18]. These spiders do not use webs to capture prey, and they use shells for building nests and shelters, where they molt, mate, oviposit, and hibernate (or are otherwise inactive) [8]. As reported by several authors, shells are extensively used for this purpose, in particular by three species of the family Salticidae: *Pellenes tripunctatus*, *Pellenes nigrociliatus*, and *Attulus penicillatus*, [18,19,20]. In addition, *P. nigrociliatus* and *P. tripunctatus* have been found to have a preference for the shell of a certain species of gastropod. *Pellenes nigrociliatus* uses shells of the gastropod *Xerolenta obvia* for reproduction and rearing of offspring [19,21] while *P. tripunctatus* most commonly uses the shells of *Caucasotachea vindobonensis* for these purposes [22]. 

Having worldwide distribution except for in South America, the genus *Pellenes* contains a large number of species (86 known to date) [23]. Nineteen of these species are known in Europe [24], and these have Eurasian distribution from Europe to China [25]. The species *P. nigrociliatus* and *P. tripunctatus* inhabit different steppe-like habitats with different affinities for the presence of rocks. *Pellenes nigrociliatus* usually prefers rockier habitats than *P. tripunctatus*, except that, on loess and sandy soils, they are often sympatric [26]. The genus *Attulus* is known in the Holarctic [23], and consists of 23 species [25]. Within that genus, only *A. penicillatus* has been recorded inhabiting shells [20]. This species has a Palearctic distribution from Europe to Japan. Within steppe-like habitats, it mainly prefers rocky places [27]. 

With some exceptions, the Salticidae are solitary species and active hunters [28,29]. Usually, random encounters end with the expulsion of one individual from the territory [30], but cannibalism is also relatively common. This phenomenon occurs in all combinations in terms of the sex of an individual, but it is more common in the interaction of two females. Cannibalism occurs both with and without certain communication signals [31,32]. 

In the case of *Phidippus audax*, *Paraphidippus marginatus* and *Marpissa undata*, an overwintering group was recorded in dense cocoons under bark. In summer, retreats under the stalks of grass were recorded in the cases of *M. radiata* and *Sitticus littoralis*, which also clustered with other species [28]. Temporary aggregations on tree crowns and under bark were also detected in the species *M. muscosa* [3]. No coexistence between groups has been reported, except for in the genera *Pellenes* and *Attulus*.

In the case of the *P. tripunctatus*, *P. nigrociliatus*, and *Attulus penicillatus*, it is unknown whether the microhabitat conditions of the environment and/or characteristics of the shell in any way affect the numbers of spiders occurring in the shells. We posed questions as to whether there were certain preferred environmental conditions or characteristics of shells that influenced shell selection as a wintering retreat, as no form of social behavior or aggregation has been reported in these species. Are certain environmental conditions or characteristics of shells preferred to such an extent that they would give rise to temporary overwintering aggregations of jumping spiders? 

## 2. Materials and Methods

### 2.1. Localities

**Figure 1:**
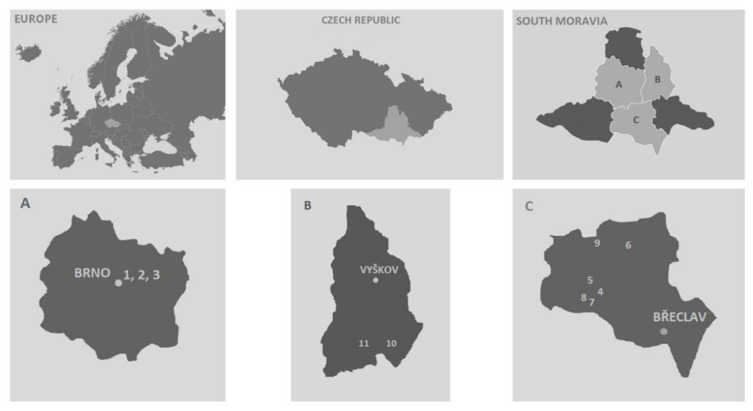
Locations of collecting areas. Area of Brno (Picture A): (1) Stránská skála, (2) Bílá hora, (3) Hády. Area of Břeclav (Picture C): (4) Milovická stráň, (5) Děvín-Kotel-Soutěska, (6) Kamenný vrch. u Kurdějova, (7) Janičův vrch, (8) Růžový kopec, (9) Pouzdřanská step. Area of Vyškov (Picture B): (10) Malhotky, (11) Člupy.

We surveyed 11 localities in the South Moravian Region within the Czech Republic during the winter of 2015/2016 for the presence of empty snail shells. The sites consisted of grassland slopes with geographic orientations to the SE, S, and SW (Table 1). All localities belonged to the Pannonian steppe region, within which there are a large number of rare and protected species. Most of the localities have certain degrees of natural conservation with ongoing management (see Table 1 and Figure 1). The entire Pannonian region is characterized by the presence of deep-horizon loess soils with individual rocky outcrops of dolomitic limestone. The whole region is generally regarded as thermophilous, and is the warmest region in the Czech Republic [33]. 

### 2.2. Field Survey

At each site, we established a transect running through the entire area of the site (11 transects). Within each transect, we searched for gastropod shells inhabited by spiders. At each finding, we placed 1 m square quadrats at contiguous meters for the full length of the linear transect, and checked the environmental characteristics for all shells contained within this square. Square meter quadrats along the transect that had no occupied shells were not assessed for environmental or shell characteristics. The number of quadrats varied among transects by shell occupancy (from 2 quadrats at Člupy and Růžový kopec to 69 at Malhotky). The collected shells belonged to the species *Caucasotachea vindobonensis* and *Xerolenta obvia*. The presence and collection of other species of gastropods was assumed. 

At each square spot, we recorded the following environmental characteristics: exposure (N, W, S, E, and combinations) and slope of the area (scaled 0–4; 0 = flat, 1 = slightly sloping, 2 = sloping, 3 = steeply sloping, 4 = very steeply sloping); management (0 = without mowing and/or grazing, 1 = mowing and/or grazing, 2 = trampling); number of shells; species of gastropod; and presence of moss, grass, herb, bare soil, and stones (scaled 0–4; 0 = without presence of the mentioned factors, 4 = substantial (approximately 80% or more) presence of the mentioned factors).

For each shell, we recorded the following shell characteristics: distance from the nearest shell (cm); damage to the shell (0 = undamaged, 1 = slightly damaged, 2 = damaged, 3 = damaged orifice); orientation of the shell aperture (1 = aperture pointing downward, 2 = aperture pointing sideways, 3 = aperture pointing upwards); the extent to which the shell was exposed below the ground (1 = shell on the surface, 2 = shell below the ground to a lesser extent, 3 = shell below the ground to a larger extent); thickness of the bands of the shell (1 = significant bands, 2 = less pronounced bands, 3 = white shells); dirtiness of the shell (1 = clean shell, 2 = slightly dirty shell, 3 = dirty shell); and the environmental conditions in the immediate vicinity of the shells at a distance of up to 5 cm (i.e., the presence of moss, stony substrate, bare soil, and vegetation (scaled 0–4, as in previous example)). 

Shells were then stored in a laboratory at about 23 °C. After a while, the spiders became active, due to increased temperature. The spiders were then inserted into test tubes with 70% alcohol, where they were fixed. The determined species were allocated to specific squares upon the transects and the shells from which they were captured.

### 2.3. Analyses

We compared the recorded presence or absence of spiders inside snail shells with the given environmental and shell characteristics. First, we analyzed the presence or absence of spiders in the snail shells against the environmental variables. The presence or absence of spiders and the spider identity were used as response variables (“species data” in the software), and environmental data were used as predictors (“environmental data”). In the next step, we exchanged the environmental data for shell characteristics. Next, we took only the shells with spider presence and compared their preference among spider species pooled according to sex. For all calculations, we used multivariate statistics in Redundancy Analysis (RDA) or Canonical Correspondence Analysis (CCA), implemented in Canoco 5, Windows release (5.10) [34], with 999 Monte Carlo permutations.

## 3. Results

The most numerous spider species were *P. tripunctatus* (56 individuals), *P. nigrociliatus* (52 individuals), and, to a lesser extent, *A. penicillatus* (11 individuals). Other spider species included *Euryopis quinqueguttata*, *Heliophanus flavipes*, and *Talavera aequipes*. Species diversity is provided in Table 2. A total of five species on the Red List of Spiders in the Czech Republic were found [35]. The species *Euryopis quinqueguttata*, *A. penicillatus*, and *Cheiracanthium pennyi* are in the endangered (EN) category. High occupancy rates of spiders were mainly recorded at Stránská skála (31.6%) and Hády (29.9%). A relatively high occupancy rate was also recorded at Děvín (20.2%).

We established 144 quadrats at 11 localities. Together, the sampled squares contained a total of 889 shells, and we documented information about environmental conditions, as well as shell characteristics, for each of them. We collected 186 shells with the presence of spider webs in the shell orifice, which totaled 20.92% of all the shells collected. However, only 113 shells (12.7% of the total) were occupied by spiders. The spiders consisted of 12 identified and 1 unidentified species (of the family Theridiidae), and totaled 146 individuals.

**Figure 2:**
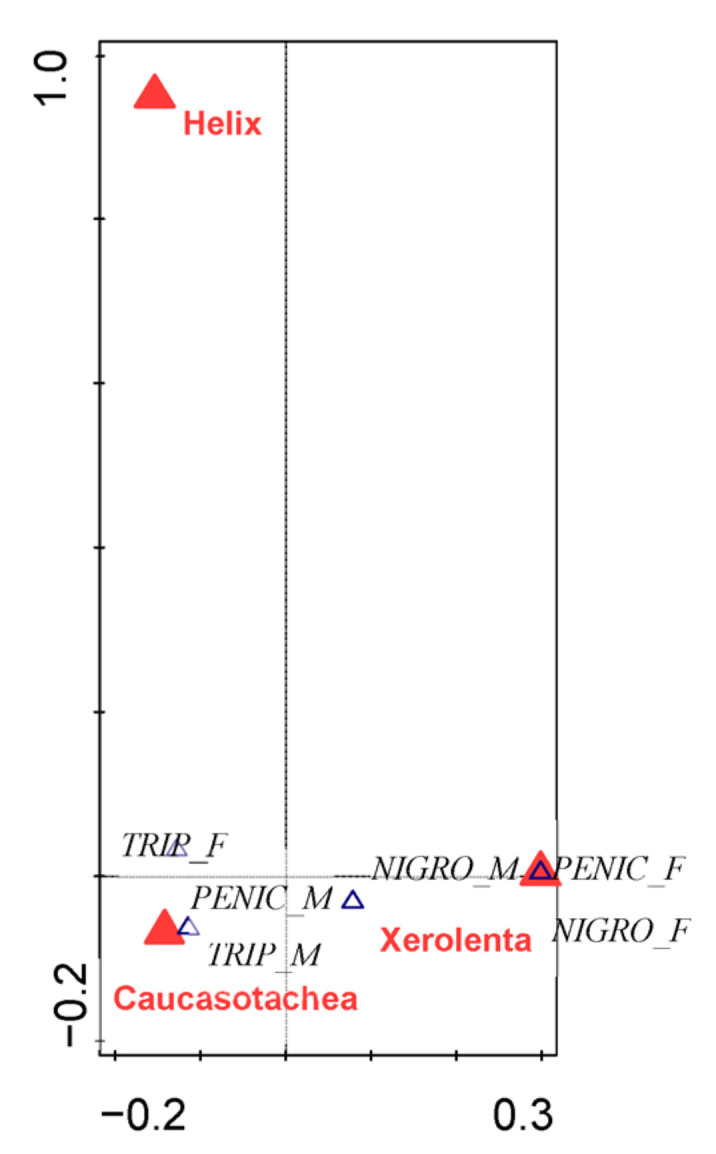
Affinities to shells of specific gastropod species: TRIP_F = *Pellenes tripunctatus* female, TRIP_M = *Pellenes tripunctatus* male, NIGRO_F = *Pellenes nigrociliatus* female, NIGRO_M = *Pellenes nigrociliatus* male, PENIC_F = *Attulus penicillatus* female.

Spider presence varied significantly among localities and between snail species (Table 3). The presence of spiders and spider species was also affected by the vegetation cover and by shell characteristics. Shell density did not affect occupancy (Table 3 and Table 4). Figure 2 describes the spiders’ affinities for the shells of certain gastropod species. *Pellenes tripunctatus* inhabited the shells of *C. vindobonensis* and, very rarely, the shells of young individuals of *Helix*. A high proportion of *Helix* shell occupancy by the females of *P. tripunctatus* was found. This information was not entirely reliable, however, due to the low number of shells collected, as just six shells of *Helix* were collected altogether, and three of these were inhabited. The reasons for their collection were the presence of spider webs in the orifices of the shells and their similarity in size to the shells of *C. vindobonensis*. The situation was clearer in the case of *P. nigrociliatus*, which had a very strong tendency to use shells of *X. obvia* to overwinter. The spiders of *A. penicillatus* chose the shells of both *X. obvia* and *C. vindobonensis*. 

Comments on the evaluation of natural conditions and shell characteristics of the microhabitats will mainly concern the species *P. tripunctatus* and *P. nigrociliatus*, due to the low numbers of *A. penicillatus* individuals acquired.

**Figure 3:**
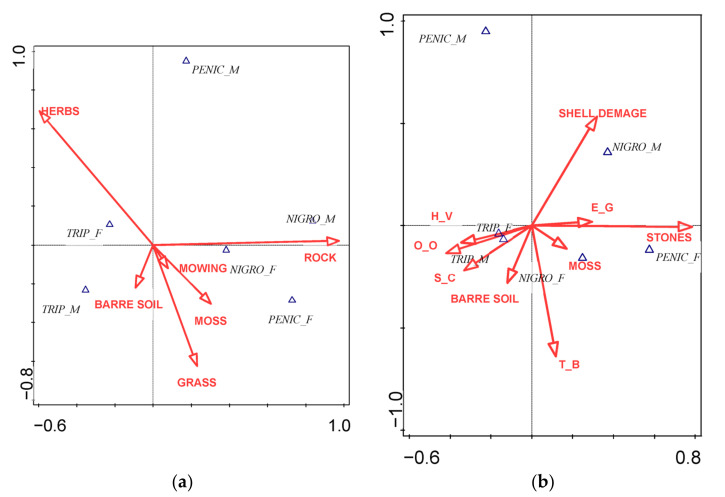
Influence of environmental conditions within the wider environment (1 × 1 m): (**a**) of immediate surroundings (**b**) of shells. H_V = Height of vegetation, O_O = Orifice orientation of shells, S_C = Shell cleanliness, T_B = Thickness of the shell bands, E_G = Exposure below the ground.

Regarding the environmental characteristics of the surrounding areas (i.e., the area of 1 × 1 m^2^), *P. nigrociliatus* preferred a rocky surface or stony substrate with a smaller proportion of moss, with preferences also differing by sex (Figure 3a). While the females preferred a larger proportion of moss, the males favored stony surfaces. Preferences were even more pronounced in the case of *P. tripunctatus*. This species searched for places with bare soil and a higher proportion of herbs, and the variation in preferences of the natural characteristics according to the sex of the individuals was much more obvious. Males preferred the presence of exposed soil with a smaller proportion of herbs. In females, a higher proportion of herbs was a priority.

A similar situation existed regarding the immediate vicinity (up to a distance of 5 cm) of occupied shells (Figure 3b). For the males of *P. nigrociliatus*, shell damage did not affect the selection of shells. The females of this species preferred shells that were surrounded by moss and stony substrate. The species *P. tripunctatus* preferred the presence of bare soil and higher vegetation. The most important factors did not differ significantly between the sexes. Occupation depended upon the direction of the aperture. Shells opening upwardly or laterally were inhabited more. Also, the cleanliness factor was of some importance. Pure or only slightly dirtied shells were inhabited more frequently.

Regarding our focal species, 18 cases of more than one overwintering specimen in a single shell were recorded, even though gregarious coexistence of these focal species had not been recorded so far. Of these, nine shells were collected at Malhotky, seven at Stránská skála, and one each at Děvín and Janičův vrch. The more common situation was overwintering of multiple individuals of one species, often males and females. Interspecific gregarious behavior was reported. Both species, *P. tripunctatus* and *P. nigrociliatus*, overwintered together with other species (see Table 5). The highest number of spiders in a single shell was six *P. tripunctatus* (five females and one male) that overwintered in a shell of *C. vindobonensis* at Malhotky.

## 4. Discussion

Our results presented interesting findings regarding the differences in environmental preferences for overwintering retreat into shells between sexes in the species *P. tripunctatus* and *P. nigrociliatus*. Females preferred more vegetation mass around the shell. In addition, 18 cases of gregarious overwintering were found, 9 of which were from the focal species. The highest number was six individuals of *P. tripunctatus* in a single shell of *C. vindobonensis*. Although several authors have dealt with the topic of spider overwintering in empty shells of gastropods [18,19,20], they have not investigated the impact of the environment or the influence of the various shell characteristics on the selection of the shell. Therefore, this work provides new and verified knowledge and facts that can substantially contribute to enriching the existing knowledge regarding this topic.

The research was carried out at localities that were selected mainly for the presence of the species of interest (*P. tripunctatus*, *P. nigrociliatus*, and *A. penicillatus*). Regarding these species, the aforementioned studies have already confirmed that *P. nigrociliatus* and *P. tripunctatus* have affinities for empty shells [19,21,22] (personal observation). Therefore, we had assumed that a large number of individuals would be acquired and that this would strengthen the usefulness of the results for examining the requirements for habitat conditions and shell characteristics. 

The densities of the empty shells at a locality played no role in the number of overwintering spiders at that locality, as was also found in the work of Bauchhenss [20]. It was enough if the preferred types of shells existed at those locations, and a smaller number of these led to gregarious hibernation. Concerning the preference for shells of certain gastropod species, the findings from previous research have been confirmed [19,21,22]. *Pellenes tripunctatus* most commonly used the shell of *C. vindobonensis* and *P. nigrociliatus* used the shell of *X. obvia*. 

The relatively low presence of spiders on mowed habitats was not surprising. The shells became visible after mowing, which means that these shells were not available earlier, because they were covered by dry litter and dead vegetation. The timing of the mowing, not taking place in the 2015/2016 winter, was relevant to our data collection. 

A very important finding was that the females of both species preferred more vegetation around the shell than did the males. It was found in the case of *P. nigrociliatus*, that a special life strategy had been created. As explained by Mikulska in 1961 [21], females hang up empty shells on vegetation using a dense web, and then create a nest for the establishment of a new generation inside the shell [21]. After hatching, the female ensures protection of her offspring. The young spiders remain in the shells until they are able to reproduce [19]. In this case, therefore, *P. nigrociliatus* females were looking for more vegetation (specifically moss), which was likely to be used in the future to hang the shell that initially was used to overwinter. There exists no record newer than Mikulska’s report from six decades ago for this unusual life strategy of hanging shells. This phenomenon was even more strongly apparent in the case of *P. tripunctatus*, for which the difference by sex in preferences for natural characteristics was much more visible. In females, a higher proportion of herbs was a priority. These data provided the first observations of hanging shells on vegetation by *P. tripunctatus* and, having observed this repeatedly at two locations, we can say this certainly appears to be a regular phenomenon in this species. Even though no work has been published to date clearly confirming the hanging of shells in the species *P. tripunctatus*, we had also documented the phenomenon at the Malhotky and Švařec localities in 2017 [36]. Females used webs to hang up empty shells on stalks of grass. In this case, a similar life strategy to that of *P. nigrociliatus* probably also applies to *P. tripunctatus*, thus creating a foundation for the future generation and rearing of offspring. Young spiders inside the hanging shells constitute a group within which, for some period of time, the individuals are relatively tolerant of one another. It is likely that a certain affinity or form of sociability is formed between the individuals during this period. It was found that the highest number of overwintering spiders recorded in one shell was six individuals of *P. tripunctatus* in a shell of *C. vindobonensis* (five females and one male). The exact record of the gregarious overwintering is described in Table 4. Another possible explanation for clustering may be that a temporary group of individuals is created at a time when aggression must be suppressed and tolerance increased [37]. In this case, communication between individuals becomes important. Even in the case of group overwintering, a certain form of communication can influence a grouping of spiders. In addition, the salticids use excellent eyesight, in addition to chemical, sound, and tactile signals [29,38]. It is possible that this could be a certain form of aggregation in which individuals lived in colonies for certain periods when they benefitted from this cohabitation [29,39]. The sub-sociality of spiders has been mainly mentioned in cases of maternal care or cooperation among young spiders in the nest after the mother’s death (especially in the families Scytodidae, Ereseidae, Amaurobiidae, and Agelenidae) [40,41,42,43,44]. In the case of the genus *Pellenes*, sub-sociality has not been reported. Among the salticid spiders overwintering in the terrestrial shells of gastropods, it is an unusual and rare situation wherein individuals are able to tolerate each other. Even in this case, the clustering has to have had certain advantages. It may have been a case of social thermoregulation inside the shell, which is common in insects [45]. This phenomenon is supported by our findings of gregarious overwintering of different species together. They simply have an advantage if, during winter, they can maintain a sufficiently higher temperature to survive the winter together. We assumed that this also could have been influenced by the shell’s natural polymorphism and the individual characteristics of a particular shell itself in *C. vindobonensis*. Darker shells and shells with more marked bands absorb more heat from the sun [46]. Although the results did not confirm this, we cannot disprove this possible cause of temporarily aggregation. Other advantages may consist of retreat against enemies [29]. Communication, and possibly some form of agreement and learned socialization within species, which has been mentioned in some of the few works concerning jumping spiders, can be cause of the aggregation [3].

The phenomenon of overwintering in shells has never been examined very deeply, and just a few studies deal with the topic at all. Such overwintering has only been reported in Europe and not in Asia and America, although the genus *Pellenes* is present there, too. The use of shells by spiders has been mentioned here and there in various places [16,18,19,20,21,22,47,48,49], but only rarely, and often coincident with other research subjects. 

The information presented here is important, not only for our knowledge regarding the behavioral ecology of the particular species, but also for nature conservation more generally, by way of conservation management practices. Based upon our data, we concluded that the presence of open, barren soil and low vegetation were very important for the presence and existence of these rare species of spiders. If we consider that even the most common spider species are somehow threatened [50], we can use these data in the management of in situ conservation. 

## Figures and Tables

**Table 1:** Information about visited localities.

Locality	GPS	Geology	Conservation Management	Elevation m a.s.l.	Number of Shells	Number of Quadrats
Stránská skála	49°11′28.600″ N, 16°40′33.264″ E	Limestone quarry	mown, grazed	255–310	95	9
Bílá hora	49°11′38.845″ N, 16°39′34.528″ E	Limestone outcrop	mown	270–299	75	6
Hády	49°13′2.122″ N, 16°40′7.623″ E	Limestone quarry	trampling	310–424	67	9
Milovická stráň	48°50′40.492″ N, 16°41′59.390″ E	Loess	grazed	200–297	8	5
Děvín-Kotel-Soutěska	48°52′3.454″ N, 16°38′50.178″ E	Limestone outcrop	grazed, mown	280–555	84	22
Kamenný vrch u Kurdějova	48°57′54.796″ N, 16°45′14.138″ E	Loess	mown, grazed	300–340	36	11
Janičův vrch	48°48′39.428″ N, 16°39′31.473″ E	Limestone quarry	trampling	264–306	186	8
Růžový kopec	48°49′16.353″ N, 16°37′28.167″ E	Loess, limestone	mown	260–280	6	2
Pouzdřanská step—Kolby	48°56′49.344″ N, 16°38′34.960″ E	Loess	mown, grazed	220–284	1	1
Malhotky	49°8′53.258″ N, 17°3′19.783″ E	Loess	mown	235–300	330	69
Člupy	49°9′7.751″ N, 16°57′27.793″ E	Loess	mown	250–300	2	2

**Table 2:** Species diversity identified in empty shells of gastropods.

Species	*Pellenes tripunctatus*	*Pellenes nigrociliatus*	*Attulus penicillatus*	*Heliophanus* sp.	*Euryopis quinqueguttata*	*Talavera aequipes*	*Heliophanus flavipes*	*Cheiracanthium pennyi*	*Neottiura bimaculata*	*Clubiona* sp.	*Haplodrassus* sp.	*Drassodes* sp.	Theridiidae	Total	Occupancy of Shells
															113/889
Number of individuals	56	52	11	7	6	6	2	1	1	1	1	1	1	146	12.71%

**Table 3:** Summary of multivariate tests analyzing presence or absence of spiders in empty shells.

Model	1st Axis		All Axes			
	Expl. Var.	Adj. Expl. Var.	F	p	F	p
Locality	5.83	4.76	5.7	0.001	5.4	0.001
Snail species	1.33	1.11	6.0	0.006	6.0	0.006
Shell density	2.13	0.65	1.4	0.262	1.4	0.262
Vegetation cover	2.19	1.52	3.3	0.007	3.3	0.007
Shell characteristics	2.89	1.89	3.0	0.003	2.9	0.003

Expl. var. = explanatory variable. Adj. expl. var. = adjusted explanatory variable.

**Table 4:** Summary of multivariate tests analyzing differences among spider species occupying shells.

Model	1st Axis				All Axes	
	Expl. Var.	Adj. Expl. Var.	F	p	F	p
Locality	25.40	20.64	3.8	0.001	5.3	0.001
Snail species	19.14	15.77	5.4	0.001	5.7	0.001
Shell density	8.26	0.00	0.4	0.661	0.5	0.761
Vegetation cover	23.98	13.61	1.4	0.001	2.3	0.003
Shell characteristics	27.53	11.63	0.8	0.002	1.7	0.004

**Table 5:** Records of gregarious overwintering.

Locality	Type of Shell	Species of Spiders				
		*P. tripunctatus*	*P. nigrociliatus*	*A. penicillatus*	Other Species of Spiders	Total
Malhotky	*Caucasotachea*	3				3
Malhotky	*Caucasotachea*	2				2
Malhotky	*Caucasotachea*	1			*Neottiura bimaculata*	2
Malhotky	*Caucasotachea*	3				3
Malhotky	*Caucasotachea*	4			*Theridiidae* sp.	5
Malhotky	*Caucasotachea*	1			*Euryopis quinqueguttata*	2
Malhotky	*Caucasotachea*	1			3× *Euryopis quinqueguttata*	4
Malhotky	*Caucasotachea*	1			2× *Euryopis quinqueguttata*	3
Malhotky	*Caucasotachea*	6				6
Stránská skála	*Xerolenta*		1		*Heliophanus flavipes*	2
Stránská skála	*Xerolenta*		2			2
Stránská skála	*Xerolenta*			1	*Talavera aequipes, Heliophanus* sp.	3
Stránská skála	*Xerolenta*			3		3
Stránská skála	*Xerolenta*		1		*Talavera aequipes*	2
Stránská skála	*Xerolenta*				2× *Heliophanus* sp.	2
Stránská skála	*Xerolenta*				2× *Heliophanus* sp.	2
Děvín	*Xerolenta*		2			2
Janičův vrch	*Xerolenta*			2		2

## Data Availability

Data are available at the Department of Zoology, Fisheries, Hydrobiology and Apiculture, with the Faculty of AgriScience, at Mendel University in Brno, Zemědělská 1, 613 00 Brno, Czech Republic.
